# Exogenous supply of Hsp47 triggers fibrillar collagen deposition in skin cell cultures in vitro

**DOI:** 10.1186/s12860-020-00267-0

**Published:** 2020-03-30

**Authors:** Essak S. Khan, Shrikrishnan Sankaran, Lorena Llontop, Aránzazu del Campo

**Affiliations:** 1 INM – Leibniz Institute for New Materials, Campus D2 2, 66123 Saarbrücken, Germany; 2 Chemistry Department, Saarland University, 66123 Saarbrücken, Germany

**Keywords:** Hsp47, Collagen deposition, Extracellular matrix, Collagen fibrils

## Abstract

**Background:**

Collagen is a structural protein that provides mechanical stability and defined architectures to skin. In collagen-based skin disorders this stability is lost, either due to mutations in collagens or in the chaperones involved in collagen assembly. This leads to chronic wounds, skin fragility, and blistering. Existing approaches to treat such conditions rely on administration of small molecules to simulate collagen production, like 4-phenylbutyrate (4-PBA) or growth factors like TGF-β. However, these molecules are not specific for collagen synthesis, and result in unsolicited side effects. Hsp47 is a collagen-specific chaperone with a major role in collagen biosynthesis. Expression levels of Hsp47 correlate with collagen deposition. This article explores the stimulation of collagen deposition by exogenously supplied Hsp47 (collagen specific chaperone) to skin cells, including specific collagen subtypes quantification.

**Results:**

Here we quantify the collagen deposition level and the types of deposited collagens after Hsp47 stimulation in different in vitro cultures of cells from human skin tissue (fibroblasts NHDF, keratinocytes HaCat and endothelial cells HDMEC) and mouse fibroblasts (L929 and MEF). We find upregulated deposition of fibrillar collagen subtypes I, III and V after Hsp47 delivery. Network collagen IV deposition was enhanced in HaCat and HDMECs, while fibril-associated collagen XII was not affected by the increased intracellular Hsp47 levels. The deposition levels of fibrillar collagen were cell-dependent i.e. Hsp47-stimulated fibroblasts deposited significantly higher amount of fibrillar collagen than Hsp47-stimulated HaCat and HDMECs.

**Conclusions:**

A 3-fold enhancement of collagen deposition was observed in fibroblasts upon repeated dosage of Hsp47 within the first 6 days of culture. Our results provide fundamental understanding towards the idea of using Hsp47 as therapeutic protein to treat collagen disorders.

## Background

Collagen (COL) fibers represent 60–80% of skin dry weight and confer skin its resistance to mechanical stress [1–4]. The skin is a layered tissue, and the collagen composition and morphology of each layer is different [5, 6]. COL I is predominant in the dermal and hypodermal layer, and forms heterotypic structures with other collagens such as COL III and/or V [7]. The basement membrane separating the epidermis and dermis is rich in COL IV. In multiple skin pathologies collagen organization is altered, either genetically or acquired due to environmental factors. Genetic collagen-related skin disorders such as Epidermolysis bullosa (EB) [8] and Ehlers-Danlos Syndrome (EDS) are both caused due to mutations in fibrillar COL I [9] and/or COL III [10]. The patients have fragile skin, blisters and chronic wounds as a consequence of reduced collagen levels in the skin tissue due to collagen misfolding, impaired formation of highly organized structures, poor collagen crosslinking, and accelerated collagen degradation [11]. Scurvy and Aging have localized wrinkles and blisters due to weakening of skin structural architecture between dermis and epidermis due to sparse collagen fiber density and extensive degradation of fibrillar collagen, mostly COL I [12, 13] by matrix metalloproteinase [14, 15]. The existing therapies for these disorders are based on the delivery of growth factors (e.g. TGF-beta [16, 17]) and chemical stimulants (e.g. ascorbic acid [17–19], glycolic acid [20], 4-phenyl butyric acid (4-PBA) [21] and retinol [22]) to boost the collagen production and matrix deposition. However, these molecules have multiple other roles in the body and the therapies are associated with negative side effects, such as promoting abnormal angiogenesis, or inflammatory responses.

We recently demonstrated that treatment of fibroblast cultures with exogenous Hsp47 specifically enhances collagen deposition [23]. Uniquely, Hsp47 is a collagen-specific chaperone. It has multiple roles in collagen biosynthesis, i.e. it stabilizes triple helical of procollagen at body temperature [24–29], it prevents intracellular procollagen degradation [30–32], it is involved in quality control of folded procollagen [32, 33], it inhibits procollagen aggregate formation in the Endoplasmic Reticulum (ER) [34, 35], and it supports procollagen transport to Golgi apparatus [31] by binding to procollagen in the ER (at neutral pH) and dissociating in the cis-Golgi (at low pH). The involvement of endogenous Hsp47 in the biosynthesis of collagen subtypes I to V has been reported [23, 30, 35, 36]. It is however unclear if exogenous administration of Hsp47 to cells affects the deposition of each collagen subtype to a similar level, or if deposition of certain collagen is preferentially supported. Note that expression levels of Hsp47 are altered in some variants of EB [37] and EDS [30] and this protein is up regulated in cancer [38–40].

Hsp47 is retained in the ER via KDEL receptor mediated transport from the Golgi to the ER [26, 30, 41–45]. This KDEL-receptor is also found on the cell membrane, [46] and is critical for the uptake of exogenous Hsp47 and transport to the ER of cells by simply adding the protein to the culture medium [23]. Expression levels of KDEL receptor at the cell membrane are cell-dependent [47, 48]. Therefore, Hsp47 uptake and the resulting up regulated collagen expression levels by exogenous Hsp47 might also be cell-dependent.

**Fig. 1:**
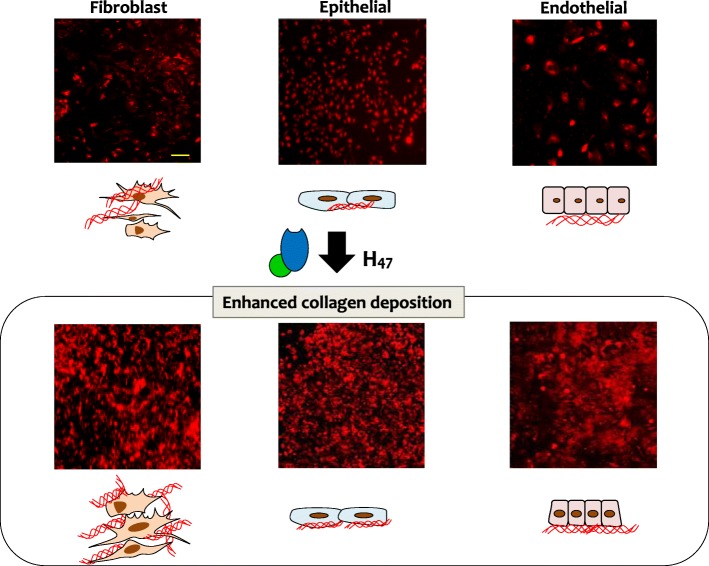
Scheme showing enhanced collagen deposition by treatment with recombinant H_47_. Immuno-staining of COL I in decellularized matrices of fibroblast, epithelial and endothelial cell line cultures is shown in Red using COL I antibody. Scale: 250 μm

In the current work we follow-up on our previous demonstration of Hsp47-stimulated deposition of collagen in fibroblast cultures [23], and we quantify the cell- and type-specific collagen deposition (fibrillar collagens I, III, V, network collagen IV, and fibril-associated collagen XII) after Hsp47 stimulation in cultures of fibroblasts, epithelial and endothelial cells from skin tissue, from human and mouse (Fig. 1). Lastly, the increase in the collagen deposition upon repetitive delivery of recombinant Hsp47 was also studied. Our results provide fundamental understanding of the potential of Hsp47 as therapeutic protein.

## Results

The uptake of Hsp47 was tested in human fibroblast (NHDF from dermis), epithelial (HaCaT, epidermal keratinocytes) and endothelial (HDMEC from dermis) skin cells. Since knocking out Hsp47 is embryonically lethal, the only established stable Hsp47 knockout cell-line currently available is a fibroblast cell line extracted from a Hsp47 knocked-out mouse embryo [49]. We used this cell line to test the activity of exogenously supplied H_47_ to trigger collagen deposition without the influence of endogenous Hsp47. We also tested other two mouse fibroblasts: MEF wild type cells from embryos and L929 (from adipose tissue) as skin fibroblast from adult mice [50], representing an intermediate model between human adult skin fibroblasts and MEF cells.

A recombinant EGFP-tagged Hsp47 [23] (hereafter referred to as H_47_) was used for these studies. The different cell types were seeded at the same cell density and incubated with H_47_ at concentrations between 0.1 μM and 1.0 μM. The incubation time of 3 h was uptaken from our previous experience [23]. H_47_ uptake was visualized by epifluorescence imaging of EGFP green signal. Incubation with H_47_ concentrations below 0.1 μM showed no detectable fluorescence signal inside the cells. Cells incubated with 1 μM or higher H_47_ showed fluorescent aggregates on the surface of the culture plate, indicating that saturation levels of H_47_ for uptake were achieved and H_47_ was binding to extracellular collagen deposited by the cells (Fig. S1). At the intermediate concentrations accumulation of green fluorescence was detected inside the cells and no aggregates were observed outside the cells, indicating efficient uptake of H_47_.

**Fig. 2:**
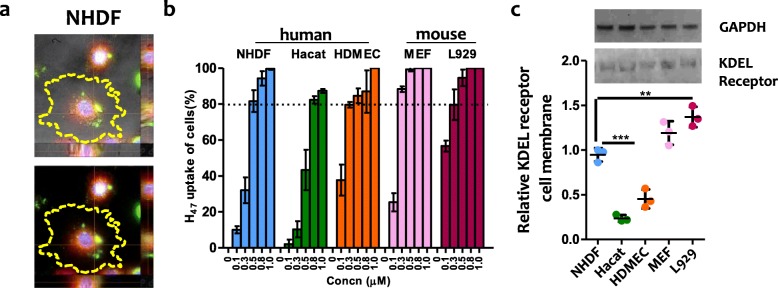
**a** Z-stack fluorescence images showing up taken 0.3 μM H_47_ (green) co-localized with ER (red) in NHDF (Blue: DAPI). The yellow dotted line around the cells indicates the edge of the cells. Scale: 20 μm, **b** H_47_ uptake by human skin cells (NHDF, HaCaT and HDMEC) and mouse fibroblast (L929, MEF) cells. Cells were incubated with increasing concentrations of H_47_ in the medium (0.1 μM–1.0 μM) for 3 h. The % of H_47_ uptake was obtained by quantifying the percentage of cells showing H_47_ (EGFP green signal) and ER tracker dye (Red signal). 100% H_47_ uptake of cells indicate that all cells in culture had co-localized signal. Error bars indicate standard deviation of *n* = 3 experiments. **c** Western blot of KDEL receptor in membrane protein fractions of human skin cells and mouse fibroblast cells using KDEL receptor antibody. GAPDH signal indicates equal cell density in cytosolic fraction of the corresponding cells stained with GAPDH antibody. Quantification data using whisker’s plot shows deviation in distribution of KDEL receptor in membrane protein fractions. Error bars indicate standard deviation of *n* = 3 experiments. Statistical significance was analyzed by Tukey test comparing KDEL receptor signal from different cell types (mean ± SD, ANOVA, *** *p* < 0.001)

Co-localization of H_47_ and the ER signals confirmed accumulation of the uptaken H_47_ at the ER in a cell types (Fig. 2a, S2). No fluorescence signal was observed when cells were incubated with EGFP alone, indicating that uptake is specific to the H_47_ sequence and not mediated by the EGFP label (Fig. S2–3). H_47_ uptake was quantified by counting the percentage of cells with detectable ER- and H_47_ fluorescent signals after incubation for 3 h and medium change. 100% uptake indicates that all the cells with labeled ER contained labeled H_47_. Results show that H_47_ uptake was concentration dependent, and also cell type dependent (Fig. 2b). An increase in the H_47_ incubation concentration led to increased uptake, and > 80% uptake was observed for fibroblasts and endothelial cells at 0.5 μM H_47_. Saturation levels are achieved at 0.5 and 0.8 μM H_47_ for MEF and L929 cells, and at 1 μM H_47_ for NHDF and HDMECs. HaCaT cells showed the lowest uptake, with maximum uptake values of approximately 80% achieved at > 0.8 μM H_47_ concentrations.

Our previous work demonstrated that H_47_ uptake occurs through KDEL receptor mediated endocytosis and transport [23] In order to check if the differences in uptake among the different cell types are related to differences in the density of KDEL receptor on the cell membrane, the membrane protein fraction from all cell types was separated and stained with KDEL receptor antibody. The cytosolic fraction was stained with GADPH antibody and taken as control for cell density. The quantified relative intensity of KDEL receptor protein bands present in membrane fraction correlated with the amount of uptaken Hsp47 in all cell types (Fig. 2 b, c). GAPDH bands were of similar intensity (0.05% variation) in all the samples, indicating similar cell density (Fig. 2 c). These results demonstrate that Hsp47 uptake by cells is dependent on KDEL receptor display on the cell membrane, as suggested in earlier reports [46].

**Fig. 3:**
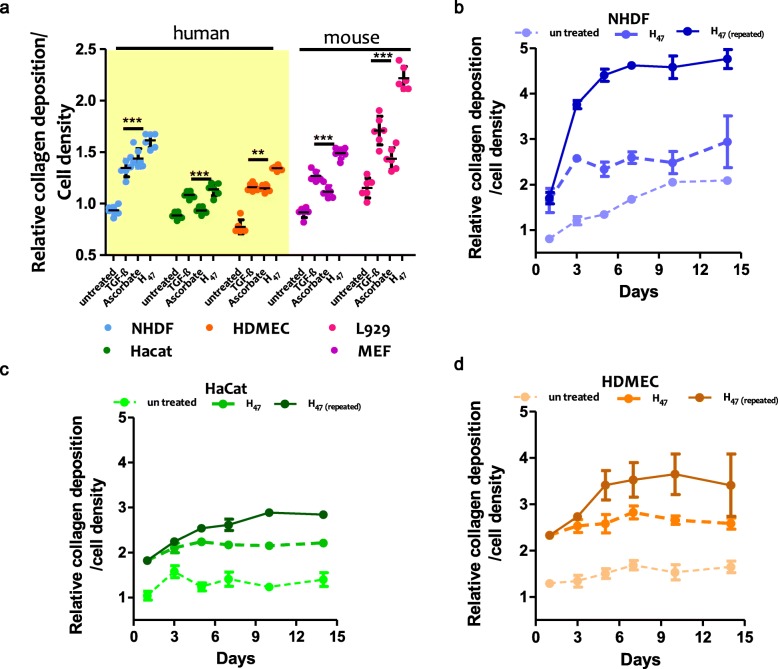
**a** Quantification of collagen deposition using Sirius Red assay in different cell types 24 h after 0.5 μM H_47_ treatment. TGF-β 1 (TGF-β) and ascorbate of same concentration are used as controls. The plots were normalized to collagen deposition in untreated NHDF. Statistical significance for both b and c was analyzed by Tukey test comparing untreated against TGF-β, ascorbate and H_47_ treated cells (mean ± SD, ANOVA, *** *p* < 0.001). Error bars representing standard deviation of 6 independent experiments. **b-d** Quantification of collagen deposition using Picro Sirius Red assay in **b.** NHDF, **c.** HaCat and **d.** HDMEC cells on days 1,3,5,7,10 and 14. Error bars represent standard deviation from 3 individual experiments

We then compared H_47_-induced collagen deposition by the different cell types after incubation with H_47_. TGF-β 1 and ascorbate were used as control collagen stimulants. Cells were seeded for 24 h on tissue culture plastic wells and incubated with 0.5 μM of H_47,_ 0.5 μM of TGF-β 1 or ascorbate for 3 h, followed by medium exchange and further culture for 24 h. The deposited collagen on the culture plate was labeled with Picro Sirius Red and quantified by spectrophotometry. Sirius red is a strong anionic dye comprising six sulfonate groups that binds preferentially to the cationic groups of the collagen fibers [51, 52]. Data were normalized to the value of collagen deposition by NHDF cells without any treatment. The human fibroblast cell line NHDF showed a 70% increases in collagen deposition when treated with H_47_, whereas the epithelial (HaCaT) and endothelial (HDMEC) cell lines showed 20 and 50% increase respectively. Interestingly, under the same conditions, fibroblasts from mouse origin (MEF and L929) also showed 70–100% increase in fibrillar collagen deposition. These data indicate that exogenously supplied H_47_ induces more effective collagen deposition in fibroblast cells. This is in agreement with the natural role of fibroblasts as major matrix-producing cells in connective tissue [53, 54]. An increase in collagen deposition was also observed in all cell types treated with TGF-β 1 and ascorbate (Fig. 3a). However, H_47_ treatment induced 20–50% higher increase in collagen deposition than the controls (Fig. 3a).

Next, we followed collagen deposition after H_47_ exposure at increasing culture times up to 14 days. Collagen deposition in fibroblasts (NHDF, MEF and L929) without H_47_ treatment increased during the first 6 days and reached a steady state at longer time scales (Fig. 3 b, S4). This trend correlates with proliferation kinetics expected for this cells, and the expected down regulation of matrix production when confluency is reached [55]. Fibroblast cells were 80–100% confluent at day 9–10. HaCaT and HDMEC cells did not show an appreciable increase in collagen deposition with time, and the overall amount of deposited collagen was much lower (Fig. 3 c, d). When cultures were treated with H_47_ on day 1, the total amount of deposited collagen doubled on day 1, at least, but the collagen deposition profile at longer times did not change significantly, i.e. the curve was just shifted to higher collagen values from day 1 (Fig. 3 b-d, S4). This result is in agreement with the expected life time of H_47_ (more than 24 h for natural Hsp47 at physiological conditions [23, 56]). These results also indicate that the deposited collagen after H_47_ treatment has a similar stability to the naturally deposited collagen without H_47_ stimulation.

We then tested if the amount of deposited collagen could be further increased by repetitive delivery of H_47_ on consecutive days. Culture medium was supplemented with 0.5 μm H_47_ on days 1, 3, 6, 9 and 12, and collagen deposition was quantified on days 1, 3, 5, 7, 10 and 14. A 2–3 fold increase in collagen deposition was observed in NHDF, MEF and L929 cells after repeated treatment with H_47_ until day 5. HaCaT and HDMEC cells showed 1–1.5 fold increase in collagen deposition within the same timescale. Addition of H_47_ on days 6, 9 and 12 did not result in further changes in the deposited amount of collagen (Fig. 3b-d, S4). This plateau of collagen deposition also occurred in the control experiments, in which cells were not treated with H_47_ within the same period of time, and is again associated with the achievement of confluency in the 2D cell culture. Confluency results in cell senescence, up-regulation of MMPs 40% (especially MMP1, 52%) and down regulation of MMP inhibitors and ECM proteins like collagens and elastin at genetic levels (approximately 4 times less) [57–60]. In order to confirm this hypothesis, H_47_ treatment was performed on 2D L929 cell cultures once they reached confluence (Day 10). No significant increase in collagen deposition was observed 24 h after this treatment (Fig. S5a), corroborating our hypothesis. In addition, H_47_ was found bound to matrix collagen on the culture substrate, with no detectable delivery inside the cells (Fig. S5b). In summary, repeated dosage of Hsp47 increased collagen production in 2D cultures of skin cells until cells reached confluency.

TGF-β and other inducers have been reported to trigger differentiation of dermal fibroblast to myofibroblast during scar formation [61, 62]. TGF-β1-induced myofibroblast differentiation through the Smad2/3 signaling pathway increases Hsp47 expression levels [63]. To verify if exogenous supply of H_47_ treatment also induced such transformation, NHDF cells were treated with both collagen stimulators and immunostained with myofibroblast transformation marker (α-SMA (α = Smooth Muscle Actin)). NHDF cultures were incubated with 0.5 μM of H_47_ and TGF-β 1 for 3 h, followed by medium exchange and 24 h cultivation. Cells were then fixed and stained with α-SMA. NHDFs treated with TGF-β 1 showed α-SMA signal, indicating myofibroblast transformation. Conversely, no α-SMA signal was observed on H_47_ treatment in NHDF cells (Fig. S6). This indicates exogenously supplied Hsp47 in controlled amount cannot trigger such side effects. These results confirm the higher specificity of the H_47_ protein towards inducible collagen deposition vs. the treatment with the growth factor, which is currently used in therapeutic treatments.

**Fig. 4:**
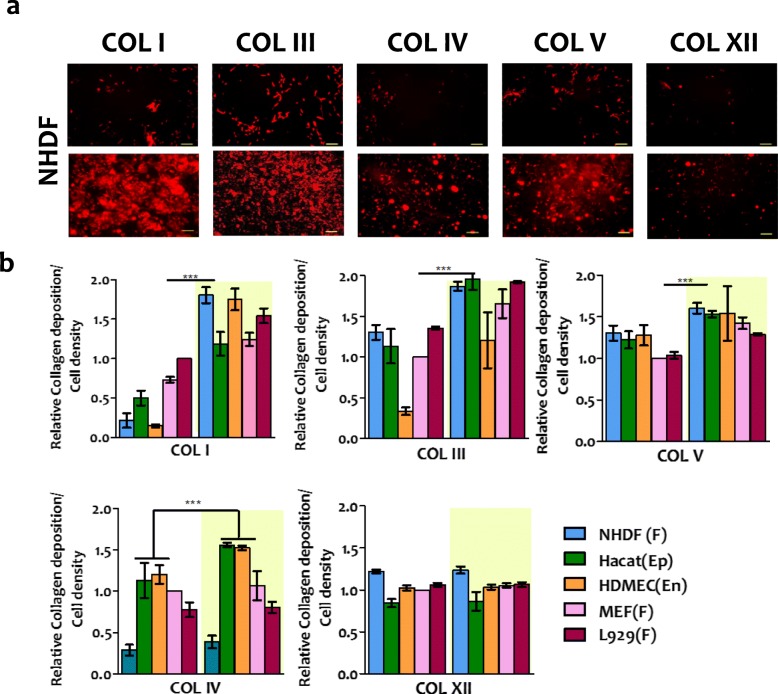
Quantification of H_47_ stimulated deposition of different collagen subtypes. **a.** Fluorescence images showing immunostained COL I, III, IV, V and XII in NHDF cells before and after treatment with 0.5 μM H_47_ (Scale – 250 μm) **b.** Quantification of deposited COL I, III, IV, V and XII from immuno-stained images in NHDF, L929, MEF, HaCaT and HDMEC cultures. Data correspond to collagen deposition 24 h after H_47_ treatment and controls. The plots were normalized with MEF cells untreated condition as 1. Error bars represent standard deviation from n-3 experiments. Statistical significance was analyzed by Tukey test comparing non treated against H_47_ treated cells (mean ± SD, ANOVA, *** *p* < 0.001)

The composition of the collagen matrix is tissue-dependent, and different cells are expected to produce different collagen types. We investigated if Hsp47-induced collagen deposited in the cell culture had the same composition as the naturally secreted collagen in the different cell types. For this purpose, H_47_-treated cultures were decellularized and the remaining matrix layer on the culture plate was stained using antibodies specific for COL I, III, IV, V and XII. These collagens were selected based on their abundance in skin tissue and their involvement in skin related disorders, in which they are reduced or mutated [9–11, 22, 54]. The relative abundance of each collagen subtype was obtained from fluorescence imaging of the culture plate. The mean fluorescence intensity value for each collagen subtype was corrected by subtraction of the background (see experimental details), and normalized by the corresponding value obtained in untreated NHDF cells. An increase in the deposition of fibrillar COLs I, III and V was observed in all cell types upon H_47_ treatment. The increase in the deposition of fibrillar collagens I, III and V was significantly higher in fibroblast cultures vs. HaCaT and HDMEC cultures. Conversely, deposition of network COL IV upon H_47_ treatment was only enhanced on HaCaT and HDMECs (Fig. 4 and S7). No changes were observed in the deposition of COL XII, suggesting that Hsp47 may not be involved in the secretion of the fibril-associated COL XII (Fig. 4b). We observed that cell spreading increased in cultures treated with Hsp47, which is an expected finding as collagen is a matrix protein with multiple adhesion sites for the integrin family.

**Fig. 5:**
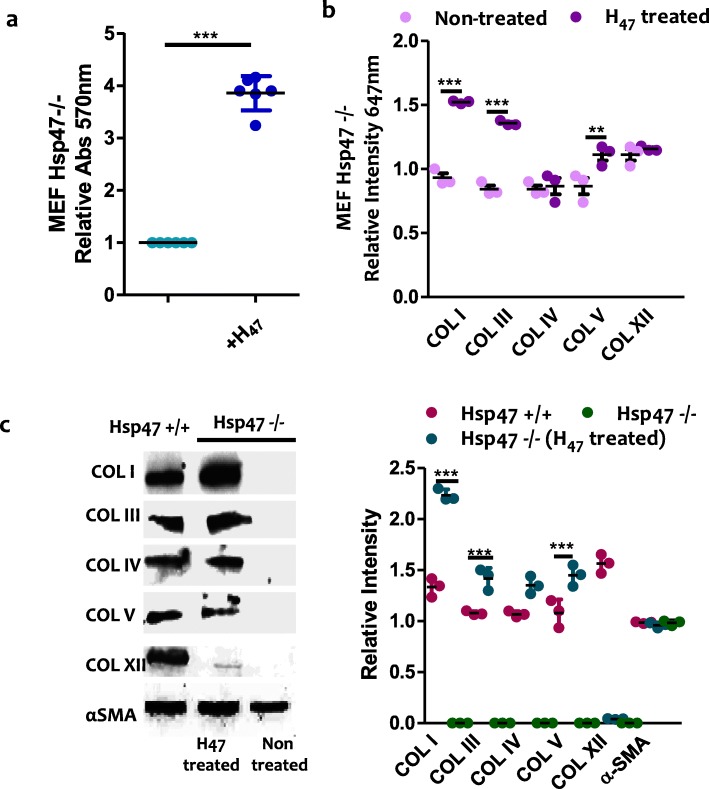
**a.** Quantification of fibrillar collagen deposited in MEF Hsp47−/− and Hsp47 +/+cells (control) at 24 h after H_47_ treatment (0.5 μM) using Sirius Red assay. **b.** Quantification of deposited COL I, III, IV, V and XII from immuno staining assays in MEF Hsp47−/− cultures. Error bars representing standard deviation from n-3 experiments in **a** and **c**, The plots in both **a** and **c** assays were normalized by MEF Hsp47−/− cells untreated condition taken as 1. Statistical significance in **a** and **c** was analyzed by Tukey test. Significance was calculated by comparing non treated against Hsp47 treated cells (mean ± SD, ANOVA, *** *p* < 0.001). **c.** Western blot of COL I, III, IV, V and XII in deposited collagen from MEF Hsp47 +/+, Hsp47 −/− and Hsp47−/− cultures 24 h after treatment with 0.5 μM H_47_. Equal amount of cells were analyzed. Black bands indicate signal from collagen subtypes specific antibody. The wiskers plot indicates relative gel bands intensity of different collagen types in MEF Hsp47 +/+, Hsp47 −/− and Hsp47−/− H_47_ treated conditions. Error bar indicates standard deviation from n-3 experiments. Statistical significance was analyzed by Tukey test. Significance was calculated by comparing non treated Hsp47 −/− against Hsp47 treated cells (mean ± SD, ANOVA, *** *p* < 0.001)

We also studied the influence of exogenously supplied H_47_ on collagen production in a previously established MEF Hsp47-knockout fibroblast cell line (Hsp47−/−), which does not produce endogenous Hsp47 [49, 64, 65]. Deposition of fibrillar COLs I, III and V increased upon H_47_ treatment. No increase in COL IV and XII was observed, in agreement with previous observations in the other fibroblast cell lines (Fig. 5 a-b, S8). In order to quantify deposited collagen subtypes at higher sensitivity, western blot analysis of the deposited matrix was performed. Higher deposition of COL I, III and V was confirmed, and a very low amount of COL IV was also detected. Interestingly, similar analysis performed with healthy MEF cells (Hsp47+/+) revealed that these cells produced these collagen types to similar extent as observed in Hsp47−/− cells after H_47_-induction (Fig. 5c). These results demonstrate that treatment of H_47_ deficient cells with exogenous H_47_ restores the ability of the cells to deposit collagen at levels and composition similar to healthy cells. Taking into account the physiological relevance of matrix composition and properties in cellular behavior in vivo we speculate that treatment with Hsp47 could be a useful approach to enhance collagen synthesis and matrix deposition on-demand.

## Discussion

Hsp47 is a collagen-specific chaperone protein with multiple roles in collagen biosynthesis. Expression levels of Hsp47 correlate with collagen production [64, 66]. Hsp47 has a regulatory role during scar formation in neonatal mouse skin after injury, which indicates that its expression during healing is up regulated in situ, in response to injury [67]. These facts indicate that Hsp47 could be an interesting therapeutic target in collagen-related skin disorders, as alternative to non-specific collagen inducers such as TGF β [68], VEGF [69] or ascorbic acid [17, 18]. Therapeutic use of these molecules to enhance collagen deposition influences other cellular functions such as proliferation [17, 18, 70, 71], differentiation [70] and angiogenesis [72], leading to undesired side effects. For example, ascorbate increases collagen production by acting as a co-factor to proline and lysine hydroxylases, which are involved in the hydroxylation of procollagen [42]. However, these enzymes are also involved in the hydroxylation of other matrix proteins, like Elastin or Fibronectin [73]. In contrast, the unique collagen-specificity of Hsp47, demonstrated in the literature [23, 30, 51], would allow up regulation of collagen deposition, without affecting any other molecule or cellular pathway.

The deposition of fibrillar collagens I and II, and to a less extent fibrillar collagen V was mainly enhanced in all tested cell types. Deposition of network COL IV was only observed in cells that are naturally connected to a basement membrane, where COL IV is also a major component. Fibril-associated COL XII was not enhanced by H_47_ treatment. The significant enhancement of fibrillar collagen deposition vs. other collagen types highlights the supporting role of Hsp47 in the intracellular assembly, stabilization and transport of collagen superstructures. A fibrillar collagen specific stimulation of collagen production could be a positive aspect for a potential Hsp47-derived therapy to enhance natural collagen production in diseases.

It is interesting to compare the efficiency of Hsp47-based enhancement in collagen deposition vs. treatment with other collagen inducers from literature data. In the comparison both the amount of deposited collagen and the time scale at which noticeable deposition occurred is relevant. We anticipate that the comparison is done among different cell types and culture methods and, therefore, numbers can only be taken as indicative. In our data, the deposition of fibrillar COLs I and III in fibroblasts (MEF, L929 or NHDF) was enhanced up to 70–100% in 24 h. In contrast, ascorbate treatment of primary healthy skin fibroblast increased collagen production by 20–40% after 4 days of treatment in vitro (measured by radioactively labeling procollagen), [74] and only 10% in Hsp47 deficient fibroblast (measured by Sirus red assay) [23]. Glycolic acid treatment increased collagen production by 48% in a week in human skin fibroblast culture from neonatal foreskin and outgrown cells [75] and Vitamin A (retinol) induced a 100% increase in chronological aging skin in human patients in vivo after 24 weeks [76]. This comparison reveals that Hsp47 promotes fast and efficient deposition of fibrillar collagen in comparison with the other molecules, which would be a beneficial feature for a therapeutic use of Hsp47.

In vivo collagen assembles into high order structures [77]. In 2D cultures the interaction of the secreted collagen with the polystyrene plate affects the assembly [78]. Studies in real tissues would be necessary to proof if the secreted collagen upon Hsp47 treatment is able to form morphologically complex structures as it occurs in the natural extracellular matrix.

## Conclusions

The collagen subtype distribution in natural tissue plays a crucial role in tissue biomechanics, and alterations result in pathological states. In the skin, alterations in collagen levels are present in EDS, EB or Scurvy, and lead to chronic wounds, blisters and skin fragility. Our results show that exogenous delivery of Hsp47 chaperone can enhance collagen deposition in a cell-specific manner. Being a collagen specific molecular chaperone, Hsp47 treatment is not expected to affect other cellular process, unlike other collagen-stimulators like ascorbic acid, glycolic acid, retinol or growth factors would do. This is a relevant advantage of Hsp47 for its potential use as matrix-stimulating therapeutic protein.

## Methods

### Synthesis and purification of H_47_

EGFP-Hsp47 (H_47_) was synthesized and purified using previously established protocol [23]. Pre-inoculums of H_47_ were grown overnight in LB medium containing NaCl (5 g/L) and kanamycin (Kan) (25 μg/ml) at 37 °C/250 rpm. After 24 h, the cultures were transferred into a 2 L flask and were grown until OD600 of 0.65 in 1 L LB NaCl Kan media at 37 °C/250 rpm for proper aeration. The protein expression was induced with 1.0 mM isopropyl-β-d-thio-galactoside and was incubated overnight at 30 °C/180 rpm. The cultured cells were harvested and pellets were stored at − 80 °C. Stored cells thawed and were resuspended in lysis buffer (300 mM NaCl, 10 mM imidazole, 20 mM Tris, pH 8) and lysed by sonication. Cleared lysate was purified by Ni-NTA affinity chromatography (Ni-NTA superflow; Qiagen). The soluble fraction was concentrated and purified further by 50 K Advanced Centrifugal Device (Macrosep) in buffer A [20 mM Hepes (pH 7.5), 300 mM NaCl, and 4 mM DTT].

### Cell culture and quantification of H_47_ uptake

Hsp47+/+ and Hsp47−/− MEFs derived from Lethal Mouse Embryos [49] were gifted by Prof. Dr. Kazuhiro Nagata, Kyoto Sangyo University, Japan, L929 fibroblasts (ATCC CRL-6364), NHDF (Promo Cell C-12302), HaCaT (ATCC® PCS-200-011) and HDMEC (Promo Cell C-12210) were purchased from commercial suppliers.

MEF, L929, NHDF and HaCaT cells were seeded on 15 well Ibidi μ-Slide Angiogenesis plates (20,000 cells per well) with DMEM GlutaMax (Gibco) containing 10% fetal bovine serum (FBS; Gibco) and 1% Penicillin-Streptomycin (Pen-strep) antibiotic. HDMEC cells (20,000 cells per well) were seeded using M199 medium with endothelial growth supplement (Gibco), 10% fetal bovine serum (FBS; Gibco) and Pen-strep antibiotic. After 24 h, cells were incubated for 3 h with varying concentrations of H_47_ (0.1 μM–1 μM). The medium was removed and cells were washed once with sterile Assay buffer (1X) provided in the kit. A dual staining solution (for nucleus and ER) was prepared by mixing 1 μl of ER tracker dye (ER Staining Kit - Red Fluorescence - Cytopainter (ab139482)) with 1 μl of DAPI in 1 ml of ER Assay buffer (1X) provided in the kit. The cells were incubated with 60 μl of Dual staining solution per well at 37 °C for 1 h. Cells were washed with Assay buffer (1X) once, fixed with 4% PFA for 10 mins and washed three times with Assay buffer (1X). All the experiments were done in triplicate.

Cell cultures were imaged using a Nikon Ti-Ecllipse microscope (Nikon Instruments Europe B.V., Germany) with a 60X objective. The number of cells showing both H_47_ signal with ER tracker signal were counted and represented in percentage. Image J was used for the analysis. Graphs were plotted using graph prism software for three independent experiments for each cell type and 10 images each.

### Quantification of KDEL receptor at the cell surface by western blotting

To quantify the density of KDEL receptor on the cell membrane a western blot analysis was performed. 2.5 million cells (NHDF, Hacat, HDMEC, MEF and L929) cells were used to extract membrane protein and cytosolic protein fraction using the Mem-PER™ Plus Membrane Protein Extraction Kit (Thermo, 89,842) following manufacturers protocol. The extracted protein fractions we mixed with 4x Lameli buffer and heated at 95 °C for 10 mins for denaturation. 15 μL of protein fractions were loaded on 12% SDS PAGE gels along with the marker. The gels were transferred to PVDF membranes using the blotting chamber. The blotted PVDF Membranes were blocked with Blocking buffer (0.5% milk powder in PBST (0.1 w/v)) for 20 min. The excess blocking buffer was washed off three times using PBST. The membrane fraction PVDF membranes were incubated for 1 h with anti-mouse KDEL receptor monoclonal primary antibody (KR-10) (Enzo life sciences, ADI-VAA-PT048-D) and cytosolic fraction with anti-rabbit GAPDH (14C10) Rabbit mAb primary antibody (Cell signaling, 2118S). Excess antibody was washed off three times using PBST (0.5 w/v) and the sample was stained with secondary antibody for 20 min at room temperature (For membrane fraction: Goat anti-Mouse IgG (H + L) Highly Cross-Adsorbed Secondary Antibody, Alexa Fluor 546, A-11030,Thermo fisher (1:500 dilution). For cytosolic fraction: (Goat anti-Rabbit IgG (H + L) Highly Cross-Adsorbed Secondary Antibody, Alexa Fluor 647, A-21245, Thermo fisher (1:500 dilution)). The PVDF membrane after staining was visualized under Gel Doc. All the experiments were done in triplicate. Image J software was used to quantify KDEL receptor signal with each band for corresponding cells and data was normalized to highest value of NHDF intensity as 1.

### Sirus red assay for quantification of collagen deposition after stimulation

Cells were cultured in 24 well plate for 24 h (50 K(50,000) cells per well) and then incubated with 0.5 μM solutions of H_47_ in DMEM and M199 medium for 3 h, followed by medium exchange and cultured for 1, 3, 5, 7, 10 and 14 days. Non treated cells were cultured for similar time points and used as controls in this experiment. For testing repeated H_47_ treatments in all the cell types, H_47_ was also added on day 1, 3, 6, 9 and 12. Collagen deposition was quantified on days 1, 3, 5, 7, 10 and 14. For the control experiments with ascorbate and TGFβ1, cells were cultured in 24 well plate and incubated with 0.5 μM ascorbic acid phosphate (Ascorbate) (Sigma, A8960-5G,L-Ascorbic acid 2-phosphate sesquimagnesium salt hydrate) and 0.5 μM TGFβ1 (Recombinant Human TGF-beta 1 Protein, 240-B-010, R & D systems) for 3 h, followed by medium exchange and cultured for 24 h.

For Sirius Red assay, cells were fixed using Bouin solution (75% picric acid, 10% formalin, and 5% acetic acid) (Sigma HT10132). Collagen deposited in the wells was stained by incubating with 0.1% Sirius red in picric acid (ab150681) for 1 h and washing with 0.01 N HCl. The matrix was dissolved in 0.1 N NaOH and the absorption of the slurry were measured at 570 nm using a Biolumin960k spectrophotometer [23, 51]. The absorbance values (Fig. 3a in manuscript) were normalized by the value of collagen deposition from untreated NHDF cells in 24 h. In MEF Hsp47 −/− untreated cells were considered as 1 for Fig. 5a.

For testing ability of H_47_ to enhance collagen deposition on cells reaching confluency, L929 were seeded at a high density (50,000 cells/ well) and allowed to become confluent within 2 days and treated with H_47_ and kept for 24 h after treatment. Collagen deposition was quantified using sirus red assay as described above. The absorbance values were normalized by the value of collagen deposition from untreated L929 cells in 24 h as 1. Also deposited collagen was stained with COL 1 antibody with the protocol mentioned below.

### Myofibroblast differentiation assay

For testing myofibroblast differentiation of NHDF cells on TGFβ and H_47_, 25,000 cells/ well were first cultured for 24 h with DMEM medium, 10% FBS and antibiotics. After this cells were supplemented with 0.5 μM of TGFβ1 (Recombinant Human TGF-beta 1 Protein, 240-B-010, R & D systems) or H_47_ in the medium. After 3 h medium was exchanged by normal medium, and cells were incubated for further 24 h. Cells were fixed with 4% PFA and stained with Anti-alpha smooth muscle Actin primary antibody (Abcam, ab5694) for 1 h at RT. Cells were washed three times with PBS and incubated with goat anti-Rabbit IgG (H + L) Highly Cross-Adsorbed Secondary Antibody, Alexa Fluor 647, Thermofischer A-21245 and DAPI (1:5000). For both primary and secondary antibodies 1:200 was used. Microscopic imaging was performed using with Nikon Ti-Eclipse microscope. All the experiments were done in triplicates.

### Immunostaining of collagen subtypes

For immunostaining the different collagen subtypes, the cultures were first decellularized using a previously established protocol [79], fixed and immunostained with collagen-specific antibodies. Cultures were treated with 0.5% Triton X-100 and 20 mM NH_4_OH for 5 min at 37 °C for decellularization, and then fixed with 4% PFA. The remaining matrix on the culture substrate was blocked with 5% goat serum in PBS and stained for COL I, III, IV, V and XII with primary antibodies as recommended by supplier (Rabbit polyclonal anti-type I collagen, 600–401–103-0.1 (Rockland), Collagen III Polyclonal Antibody (Thermo fisher, PA5–34787), Anti-COL4A3 antibody (Sigma, HPA042064-100UL), Anti-Collagen V antibody (ab7046), Anti-COL12A1 antibody (Sigma, HPA070695) (dilution 1:200 for all the antibodies)). Samples were washed 3 times with PBS and stained with secondary antibody (goat anti-Rabbit IgG (H + L) Highly Cross-Adsorbed Secondary Antibody, Alexa Fluor 647, A-21245 (dilution 1:200)). For analysis, the mean gray value of fluorescence intensity was measured for each collagen subtypes and subtracted from background mean gray value of fluorescence intensity by imaging stained collagen with only secondary antibody. Results were normalized by taking untreated MEF cells as 1. In MEF Hsp47 −/− untreated cells were considered as 1. This analysis was performed in Image J. Ten images from each condition were used for the analysis from three independent experiments.

### Quantification of collagen subtypes by western blotting for MEF Hsp47 −/−

For quantification of the collagen subtypes after H_47_ treatment, MEF Hsp47 −/− cells 50,000 cells were used as equal seeding density in all cell types. Deposited collagen from cells cultured in the presence and in the absence of H_47_ was suspended in 300 μL of RIPA Buffer. Before this step, the collagen deposited was decellularized using the above-mentioned protocol. Protease inhibitor and Lameli buffer (4x stock concentration) was added in mixture to avoid protein degradation. The samples were loaded into SDS-PAGE gels. The 12% SDS PAGE gels were transferred using blotting chamber to PVDF membranes. The Blotted PVDF Membranes were blocked with Blocking buffer (0.5% milk powder in PBST (0.1 w/v)) for 20 mins. The excess blocking buffer was washed off three times using PBST. The PVDF membranes were incubated overnight at 4 °C with collagen subtypes antibodies mentioned above. For α-Smooth Muscle (α-SMA) condition cells were not decellularized and antibodies used were Anti-Actin, α-Smooth Muscle - Cy3 (C6198). On the following day the excess was washed off three times using PBST (0.5 w/v) and the sample was stained with secondary antibody for 1 h at room temperature (Goat anti-Rabbit IgG (H + L) Highly Cross-Adsorbed Secondary Antibody, Alexa Fluor 647, A-21245 (1:500 dilution). The PVDF membrane after staining was visualized under Gel Doc. All the experiments were done in triplicate.

### Statistical significance

For Sirus Red assay (*n* = 6) number of experiment were performed and plotted with graphs including whisker plots representing standard deviation for MEF, L929, NHDF, HTCAT and HDMEC. For MEF Hsp47 −/− three independent experiments were performed. For immunostaining quantification IMAGE J was used to quantify mean intensity profile of stained matrices in all cell types treated with and without H_47_ with bar plots including error bars indicating standard deviation. In both assays the data were normalized with respect to collagen deposited in MEF untreated as 1. In case of MEF Hsp47 −/− untreated cells were considered as 1. Statistical significance was analyzed by Tukey test, which shows significant differences between conditions. Significance was calculated by comparing non treated vs treated cells (mean ± SD, ANOVA, *** *p* < 0.001).

## Supplementary information

**Additional file 1.** Figure S1 shows aggregation on H_47_ on substrate at 1 μM concentration in NHDF, Hacat and HDMEC cells.

**Additional file 2.** Figure S2 shows supplementary information on delivery of H_47_ to ER via KDEL receptor-mediated endocytosis to different cell types.

**Additional file 3.** Figure S3 shows Z-stack orthogonal projection images of NHDF after incubation with EGFP for 3 h.

**Additional file 4.** Figure S4 shows quantification of collagen deposition using Picro Sirius Red assay in a. MEF and b. L929, cells on 1,3,5,7,10 and 14 days.

**Additional file 5.** Figure S5 shows H_47_ binds to collagen on the matrix on L929 cells reaching confluency.

**Additional file 6.** Figure S6 shows immunofluorescence images of NHDF cells stained with myofibroblast marker (α SMA) on treatment of H47 and TGF-β.

**Additional file 7.** Figure S7 shows Immunostaining of COL I, III, IV, V and XII deposited in MEF, L929, HaCaT and HDMEC cultures 24 h with and without treatment of H_47_.

**Additional file 8.** Figure S8 shows Stimulated deposition of COL I, III and V in MEF Hsp47 −/− cells after H_47_ uptake.

## Data Availability

All data generated or analyzed during this study are included in this published article and its supplementary information files.

## Abbreviations

Hsp47: Heat shock protein 47
TGF-β: Transforming growth factor beta
MMP: Matrix metalloproteinase
NHDF: Normal Human Dermal Fibroblasts
MEF: Mouse Embryonic Fibroblasts
GAPDH: Glyceraldehyde 3-phosphate dehydrogenase
