# Supplementary material for: Exogenous supply of Hsp47 triggers fibrillar collagen deposition in skin cell cultures in vitro
Source: BMC Mol Cell Biol. 2020 Mar 30;21:22. doi: 10.1186/s12860-020-00267-0 (PMC7106624; doi:10.1186/s12860-020-00267-0)
Supplement: Supplementary file 1 — Additional file 1. Figure S1 shows aggregation on H47 on substrate at 1 μM concentration in NHDF, Hacat and HDMEC cells. [file 12860_2020_267_MOESM1_ESM.docx]

**Figures:**

**Figure S1.** NHDF, Hacat and HDMEC cells incubated with 1 µM H47 (green) and stained with ER tracker dye in red showing aggregates of H47 on the substrate. Scale: 50 µm.
