# Supplementary material for: Exogenous supply of Hsp47 triggers fibrillar collagen deposition in skin cell cultures in vitro
Source: BMC Mol Cell Biol. 2020 Mar 30;21:22. doi: 10.1186/s12860-020-00267-0 (PMC7106624; doi:10.1186/s12860-020-00267-0)
Supplement: Supplementary file 2 — Additional file 2. Figure S2 shows supplementary information on delivery of H47 to ER via KDEL receptor-mediated endocytosis to different cell types. [file 12860_2020_267_MOESM2_ESM.docx]

**Figure S2. Delivery of H_47_ to ER of L929, MEF, HaCaT and HDMEC cells.** Z-stack orthogonal projection images of these cells after incubation with 0.3 µM H_47_ for 3 h. Images show co localization of H_47_ and ER signals. (Blue: DAPI (Nucleus), Green: (Hsp47), and Red: ER tracker dye). Left side: EGFP treated cell lines. Right side: H_47_ treated cell lines. Scale: 20 µm.
