# Supplementary material for: Exogenous supply of Hsp47 triggers fibrillar collagen deposition in skin cell cultures in vitro
Source: BMC Mol Cell Biol. 2020 Mar 30;21:22. doi: 10.1186/s12860-020-00267-0 (PMC7106624; doi:10.1186/s12860-020-00267-0)
Supplement: Supplementary file 3 — Additional file 3. Figure S3 shows Z-stack orthogonal projection images of NHDF after incubation with EGFP for 3 h. [file 12860_2020_267_MOESM3_ESM.docx]

**Figure S3. a.** Z-stack orthogonal projection images of NHDF after incubation with EGFP for 3 h. Image show ER signal. (Blue: DAPI (Nucleus), and Red: ER tracker dye). Scale: 20 µm.
