# Supplementary material for: Exogenous supply of Hsp47 triggers fibrillar collagen deposition in skin cell cultures in vitro
Source: BMC Mol Cell Biol. 2020 Mar 30;21:22. doi: 10.1186/s12860-020-00267-0 (PMC7106624; doi:10.1186/s12860-020-00267-0)
Supplement: Supplementary file 4 — Additional file 4. Figure S4 shows quantification of collagen deposition using Picro Sirius Red assay in a. MEF and b. L929, cells on 1,3,5,7,10 and 14 days. [file 12860_2020_267_MOESM4_ESM.docx]

**Figure S4.** Quantification of collagen deposition using Picro Sirius Red assay in a. MEF and b. L929, cells on days 1,3,5,7,10 and 14. Error bars represent standard deviation from 3 individual experiments.
