# Supplementary material for: Exogenous supply of Hsp47 triggers fibrillar collagen deposition in skin cell cultures in vitro
Source: BMC Mol Cell Biol. 2020 Mar 30;21:22. doi: 10.1186/s12860-020-00267-0 (PMC7106624; doi:10.1186/s12860-020-00267-0)
Supplement: Supplementary file 5 — Additional file 5. Figure S5 shows H47 binds to collagen on the matrix on L929 cells reaching confluency. [file 12860_2020_267_MOESM5_ESM.docx]

**Figure S5. H_47_ binds to collagen on the matrix of L929 cells reaching confluency**

a. Fluorescence images of immunostained COL I (Red signal) of confluent L929 cells treated with and without H_47_ (Green signal) (Scale: 250µm). Substrates have been decellularized. b. Quantification of collagen deposition using Sirius Red assay with error bars representing standard deviation of 3 independent experiments after 24h of H_47_ treatment of confluent L929 cells. The plots in assays were normalized with untreated L929 condition as 1. Statistical significance for was analyzed by t-test test comparing untreated against H_47_ treated conditions (mean±SD, ***  p value = 0.0558) (ns=no significance).
