# Supplementary material for: Exogenous supply of Hsp47 triggers fibrillar collagen deposition in skin cell cultures in vitro
Source: BMC Mol Cell Biol. 2020 Mar 30;21:22. doi: 10.1186/s12860-020-00267-0 (PMC7106624; doi:10.1186/s12860-020-00267-0)
Supplement: Supplementary file 6 — Additional file 6. Figure S6 shows immunofluorescence images of NHDF cells stained with myofibroblast marker (α SMA) on treatment of H47 and TGF-β. [file 12860_2020_267_MOESM6_ESM.docx]

**Figure 6.** Immunofluorescence images showing myofibroblast marker (α SMA) in Red and DAPI in Blue in 0.5 µM TGF-β treated NHDF cells. No α SMA signal is observed in control and 0.5 µM H47 treated cells. Scale-20 µm.
