# Supplementary material for: Exogenous supply of Hsp47 triggers fibrillar collagen deposition in skin cell cultures in vitro
Source: BMC Mol Cell Biol. 2020 Mar 30;21:22. doi: 10.1186/s12860-020-00267-0 (PMC7106624; doi:10.1186/s12860-020-00267-0)
Supplement: Supplementary file 7 — Additional file 7. Figure S7 shows Immunostaining of COL I, III, IV, V and XII deposited in MEF, L929, HaCaT and HDMEC cultures 24 h with and without treatment of H47. [file 12860_2020_267_MOESM7_ESM.docx]

**Figure S7. Immunostaining of deposited COL I, III, IV, V and XII** on culture plates from MEF, L929, HaCaT and HDMEC cultures, 24 h after either no treatment or treatment with H_47_. Substrates have been decellularized. (Scale corresponds to 250µm).
