# Supplementary material for: Exogenous supply of Hsp47 triggers fibrillar collagen deposition in skin cell cultures in vitro
Source: BMC Mol Cell Biol. 2020 Mar 30;21:22. doi: 10.1186/s12860-020-00267-0 (PMC7106624; doi:10.1186/s12860-020-00267-0)
Supplement: Supplementary file 8 — Additional file 8. Figure S8 shows Stimulated deposition of COL I, III and V in MEF Hsp47 −/− cells after H47 uptake. [file 12860_2020_267_MOESM8_ESM.docx]

**Figure S8. Stimulated deposition of COL I, III and V in MEF Hsp47 -/- cells after H_47_ uptake**. Immunostaining of deposited COL I, III, IV, V and XII on culture plates from MEF Hsp47 -/- cells, 24 h after either no treatment or treatment with H47. Substrates have been decellularized. Scale- 250 µm.
